# Triptorelin therapy for lower urinary tract symptoms (LUTS) in prostate cancer patients: A systematic meta‐analysis

**DOI:** 10.1002/bco2.292

**Published:** 2023-10-10

**Authors:** Ravina Barrett, Brian Birch

**Affiliations:** ^1^ School of Applied Sciences University of Brighton Brighton UK; ^2^ University Hospital Southampton NHS Foundation Trust Southampton UK; ^3^ School of Medicine University of Southampton Southampton UK

**Keywords:** gonadotropin‐releasing hormone, humans, lower urinary tract symptoms, male, observational studies as topic, polyuria, prostate cancer, prostatic neoplasms, PubMed, quality of life, Triptorelin

## Abstract

**Objective:**

This systematic meta‐analysis aimed to assess the effectiveness of triptorelin therapy in reducing lower urinary tract symptoms (LUTS) in men with prostate cancer (PCa).

**Methods:**

The Preferred Reporting Items for Systematic Reviews and Meta‐Analyses (PRISMA) guidelines were followed. PubMed, Web of Science and EMBASE databases were searched for studies conducted between 2013 and 2023. Eligible studies included PCa patients undergoing androgen deprivation therapy (ADT) with triptorelin, with reported baseline and follow‐up International Prostate Symptom Scores (IPSS) and quality of life (QoL) data. The Newcastle–Ottawa Scale (NOS) was used to assess the risk of bias, and a random‐effects model was applied for the meta‐analysis.

**Results:**

A total of 29 articles were identified, and three studies met the inclusion criteria. Triptorelin therapy showed a clinically significant reduction in IPSS over 48 weeks in PCa patients with moderate to severe LUTS. The meta‐analysis revealed a pooled effect size of 1.05 (95% CI: 0.65; 1.45), indicating a statistically significant improvement in LUTS. QoL also improved in patients receiving triptorelin therapy, although heterogeneity among the studies and a moderate to high risk of bias were noted.

**Conclusion:**

Triptorelin therapy demonstrated a positive impact on LUTS in PCa patients. The meta‐analysis showed significant reductions in IPSS scores and improved QoL after 48 weeks of triptorelin treatment. However, the results should be interpreted cautiously due to study heterogeneity and potential biases. Further well‐designed studies are needed to confirm these findings and determine the optimal use of triptorelin for managing LUTS in men with PCa.

**Implications for Practice:**

Triptorelin therapy may offer an effective treatment option for men with PCa experiencing moderate to severe LUTS. Its positive impact on QoL can lead to improved patient well‐being and treatment adherence. Clinicians should consider triptorelin as a potential treatment choice, especially in patients who may be reluctant to undergo surgical interventions for their LUTS. However, careful patient selection and close monitoring are essential due to the observed study heterogeneity and risk of bias. Future research should focus on evaluating triptorelin's cost‐effectiveness and comparing its efficacy with other LH‐RH agonists in managing LUTS in PCa patients.

Video Abstract: URL (Reviewers/Editors to select from) Link 1: https://brighton.cloud.panopto.eu/Panopto/Pages/Viewer.aspx?id=071419c8-1ad5-4502-a222-b04300c2ca5e

Link 2: https://brighton.cloud.panopto.eu/Panopto/Pages/Viewer.aspx?id=b6305a8a-b977-4fcd-a69e-b04300bed728

## 1 INTRODUCTION

Prostate cancer (PCa) is the most common cancer in males in the United Kingdom, accounting for approximately 27% of all newly diagnosed cancer cases in men in 2019.^1^ Age is a significant risk factor for PCa, with approximately 34% of all new PCa cases in the United Kingdom being diagnosed in men aged 75 and over (2016–2018).^1^ This demographic shift may affect healthcare resource allocation and the development of treatment strategies.

In the United Kingdom, the incidence rate of PCa diagnosis has increased from 180.9 per 100 000 males in 2010 to 191.0 per 100 000 males in 2019.^2^ This rise is largely attributed to the increased prevalence of PSA testing, allowing for earlier detection. If the age‐adjusted PSA level is elevated,^3^, ^4^ this is considered abnormal and may indicate an increased risk of PCa^5^ should triggering a referral for further investigation.

LH‐RH agonists such as triptorelin are used to treat men with locally advanced or metastatic PCa.^6^ These agonists suppress the production of testosterone, which, at low concentrations, acts as growth factor for PCa. Triptorelin is considered the most potent LH‐RH agonist achieving the lowest mean testosterone levels and demonstrating effectiveness as an androgen deprivation therapy (ADT) for PCa.^7^, ^8^

Lower urinary tract symptoms (LUTS) encompass a range of symptoms affecting the bladder, prostate and urethra. Men with LUTS may experience, inter alia, storage symptoms (e.g. nocturia, frequency, urgency) and voiding symptoms (e.g. hesitancy, intermittency, weak stream).^9^ LUTS are common in men with PCa, which may exacerbate bladder outflow obstruction.^10^ Current treatment options for LUTS include alpha blockers (e.g. alfuzosin, tamsulosin), 5‐α‐reductase inhibitors (e.g. finasteride) and phosphodiesterase type 5 inhibitors.^11^

LUTS pose a significant burden on aging men, as their prevalence increases with age.^12^ Over time, symptoms worsen, impacting QoL.^13^ In the European Prospective Investigation into Cancer and Nutrition (EPIC) study,^14^ a multinational population‐based survey conducted in Canada, Germany, Italy, Sweden and the United Kingdom in 2005, 62.5% of men reported at least one LUTS, and more than 6% of men reported having moderate to severe LUTS. These bothersome symptoms have socio‐economic implications, causing reduced productivity, missed workdays and limited social engagement. Thus, the fact that men with PCa experience LUTS is understandable. Addressing LUTS in this population is important to alleviate symptom burden on individuals and society, as improved management can enhance QoL, reduce healthcare costs and enable individuals to maintain active and productive lifestyles.

Studies have shown LH‐RH agonists to improve LUTS in men with PCa.^15^, ^16^ Washino et al.^17^ found that the LH‐RH agonist goserelin provided notable improvements by decreasing testosterone levels and reductions in prostate gland size.^17^

The International Prostate Symptom Score (IPSS), a self‐administered questionnaire,^18^ is a validated tool widely^19^, ^20^, ^21^, ^22^, ^23^, ^24^ used in clinical practice and research to assess LUTS severity and its impact on QoL. It includes seven questions and was later modified with the addition of an eighth question on QoL. The IPSS enables consistent comparisons of symptom severity across different populations over time. Patients can complete the questionnaire independently or with healthcare professionals. While there are no specific guidelines, cohort studies typically collect IPSS data at baseline and regular intervals during follow‐up visits (e.g. 12, 24 and 48 weeks).

IPSS scores range from 0 to 5 per question, and the total score ranges from 0 to 35. Higher scores indicate more severe symptoms. Thus an IPSS score of 1–7 represents mild symptoms, 8–19 moderate symptoms, and 20–35 severe symptoms.^25^ The IPSS includes an additional question that assesses the impact of urinary symptoms on the QoL of patients. This question is scored from 0 to 6, with higher scores indicating a greater impact on QoL. The response options range from ‘delighted’ (score = 0) to ‘terrible’ (score = 6).^17^

Studies have shown the IPSS score to be a highly sensitive and specific tool that can be used to follow treatment efficacy for PCa patients.^26^, ^27^ The IPSS has limitations as it depends on self‐reported symptoms, which can be influenced by individual bias and perception.^28^ Additionally, the underlying cause of LUTS is not specifically addressed by the IPSS questionnaire; therefore, it is unable to differentiate between various LUTS aetiologies.^29^ This lack of specificity may compromise the accuracy of LUTS diagnosis and treatment. The present study systematically reviews the literature on the effectiveness of the LH‐RH agonist triptorelin in PCa patients with LUTS, addressing the need to alleviate urinary symptoms and improve patient QoL in this specific population.

## 2 METHODS

This systematic review was performed according to the ‘Preferred Reporting Items for Systematic Reviews and Meta‐Analysis (PRISMA)’ guidelines and the PRISMA checklist had been attached to supplemental material (Data S3 and S4).^30^, ^31^ Studies reporting on the effect of triptorelin in PCa patients with LUTS were searched. Collected data were then examined to evaluate IPSS and changes on QoL.

### 2.1 Search strategy

Studies were identified by electronic search of PubMed, Web of Science and EMBASE databases through a 10‐year period (28 March 2013 until 28 March 2023) by a search conducted on 28 March 2023. A manual search was also conducted by cross‐referencing relevant articles. The language was restricted to English, and only human studies were selected. The search strategy was developed focusing on PCa patients using triptorelin therapy and the effects on LUTS. The search terms in the various databases are listed in Data S1.

### 2.2 Eligibility criteria

Included studies met the following inclusion criteria:

1. Population of men with PCa who experience LUTS;
2. Recruited PCa patients who underwent ADT with triptorelin;
3. Reported patient baseline and follow‐up IPSS scores;
4. Reported cohort studies;
5. Restricted to English language articles;
6. Included outcomes of interest: effect of triptorelin therapy on total IPSS and on patient QoL;
7. A minimum participant sample size of 10, for a more accurate representation of the study population;
8. Availability of full‐text articles.

Studies were excluded if they:

1. Did not assess changes in LUTS over 48 weeks;
2. Recruited duplicates or overlapping patients from other studies;
3. Were not available as full‐text articles;
4. Did not provide original clinical data, for example, reviews, meta‐analysis, expert opinions, editorials, conference abstracts or books.

### 2.3 Data extraction

Relevant search records were extracted and imported into EndNote referencing software,^32^ followed by filtering out duplicate records. The titles and abstracts of the remaining records were screened independently by the authors against the eligibility criteria. Studies that met the criteria in the initial screening were retrieved in full text and assessed for final inclusion. Missing and ambiguous data are described. Multiple imputation was considered but decided against because greater than 10% of data were missing for the IPSS and QoL scores. Where there was a conflict of opinion, an independent third party provided resolution.

### 2.4 Quality assessment and risk of bias

The Newcastle–Ottawa Scale (NOS) for observational cohort studies was used to assess the risk of bias.^33^ This scale assesses studies using criteria including selection, comparability and outcome. Study ratings range from 0 to 9, with scores of 0–2 indicating poor quality, scores of 3–5 indicating fair quality and scores of 6–9 indicating good or high quality.

### 2.5 Meta‐analytical method

A meta‐analysis was considered and appropriately investigated^34^, ^35^ including the use of statistical software.^36^ The data were initially appraised for heterogeneity which was found to be acceptable (*I* ^2^ = 87%); hence the random‐effects model was selected. Details on meta‐analytical method included inverse variance method, restricted maximum‐likelihood estimator for 𝜏^2^ and Q‐Profile method for confidence interval of 𝜏^2^ and 𝜏. Analysis was done using RStudio using the ‘within‐group standardised mean‐difference formula’^37^ (see Data S2).

## 3 RESULTS

**FIGURE 1:**
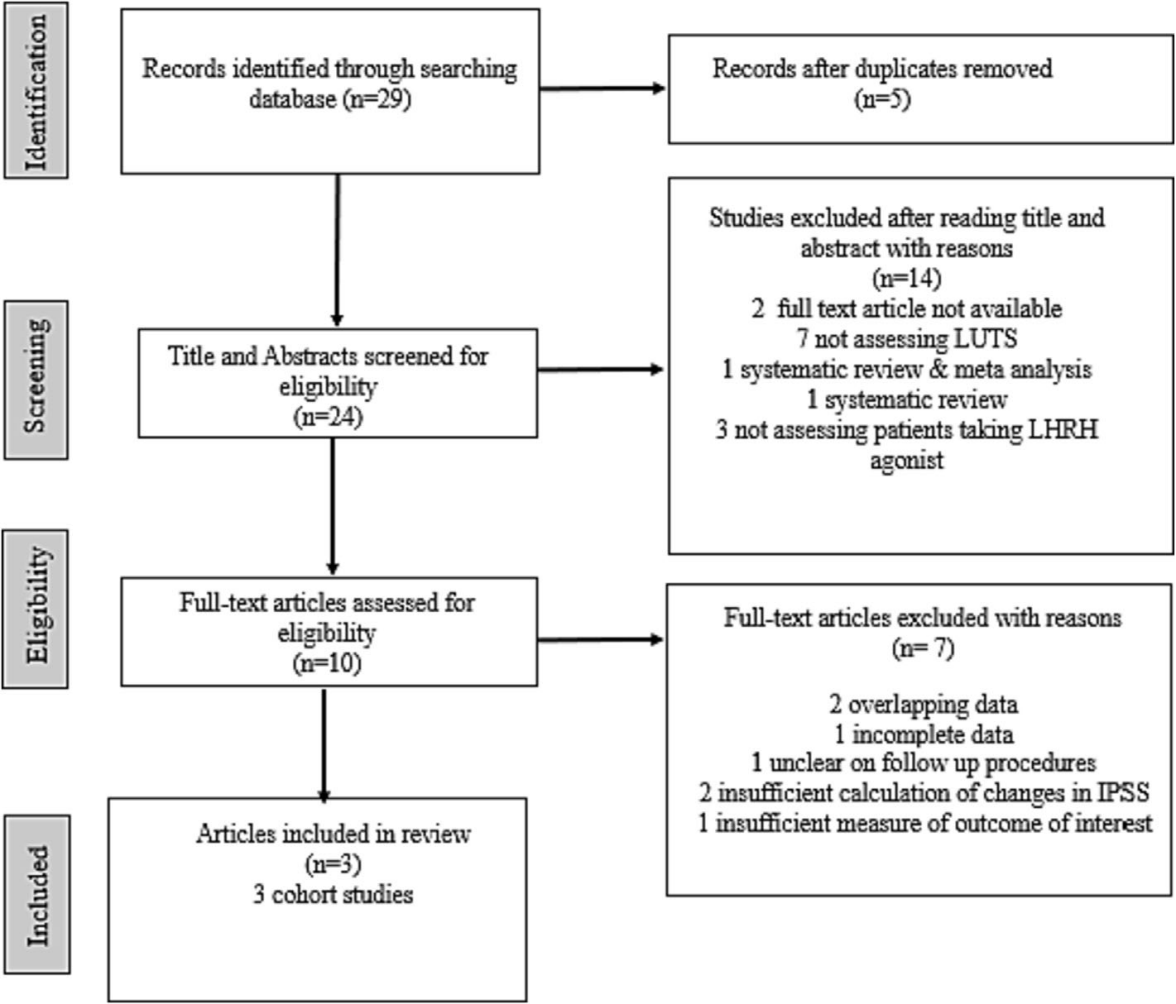
Flow diagram describing the study selection process for the systematic Meta‐Analysis methodology as per PRISMA 2020 guidelines.^31^

The flow diagram in Figure 1 shows the results of the screening and selection process. A total of 29 articles were identified from Embase, PubMed and Web of Science databases. After removing five duplicate studies and excluding 14 studies evaluated by title and abstract, full‐text articles from the remaining 10 studies were examined. Following this, a further seven studies were excluded for the following reasons: Two articles included patients with overlapping data,^10^, ^38^ making it difficult to distinguish overlap; one study had incomplete data and was stopped early due to recruitment difficulties^16^; one study was unclear on follow‐up procedures^39^; two studies had insufficient data to calculate the changes in IPSS over time^17^, ^40^; and one study, while having IPSS and QoL scores, did not specify which LH‐RH agent was under study.^15^ Finally, three studies remained eligible for inclusion.^41^, ^42^, ^43^

### 3.1 Overview of the included studies

The studies included in this meta‐analysis all had data at the time points specified (i.e. baseline and 48 weeks) and investigated the effectiveness of triptorelin therapy in reducing total IPSS in PCa patients with moderate or severe LUTS. Patients were followed up for 48 weeks after initiating triptorelin and attended three study visits: Visit 1 was at baseline, visit 2 was at 24 weeks from baseline, and visit 3 was at 48 weeks from baseline. The included studies failed to recruit the intended number of patients and closed to recruitment earlier, but were in length, between 7 and 10 years from study start to publication.

A summary of the patient and treatment characteristics and the main findings are shown in Table 1. He et al. recruited 398 patients,^41^ Peltier et al. enrolled 325 patients,^42^ and Woo et al. enrolled 44 patients^43^ accounting for the total of 767 PCa patients included in this review. The estimated mean age of patients in the three studies was >72 years (see Data S1). For a period of 48 weeks, each study reported as follows: He et al. treated patients with 15 mg triptorelin every 12 weeks, Peltier et al. treated patients with either 11.25 mg triptorelin every 12 weeks or 3.75 mg triptorelin every 4 weeks, and Woo et al. treated patients with 11.25 mg triptorelin every 12 weeks. An IPSS change of more than 3 points from the baseline was determined to be a clinically significant response in the included studies and is supported by Morote et al.^15^ Woo et al. and Peltier et al. were emailed for further clarification, but no reply received.

**TABLE 1:** Baseline characteristics of studies included in this review (SD: standard deviation).

Author, year, region	Study design	Enrolled patients (n)	Full analysis population of PCa patients with moderate and severe LUTS (n)	Excluded/Lost to Follow‐up after enrolment	Mean age at Baseline	Mean weight at Baseline	TNM at Baseline 1	TNM at Baseline 2	Mean Gleason score at Baseline	Mean Baseline PSA, ng/mL	Mean PSA, ng/mL (At 48 weeks)	Main findings: Total IPSS at baseline	Main findings: Total IPSS (At week 48)	Main findings:Total change in IPSS from baseline (At week 48)	Triptorelin Dosage of LH‐RH agonist triptorelin	Follow‐up time (weeks)
Le‐Ye He, 2018, China	Cohort (prospective)	398	255	143	72.2 years	65.9 kg	T3N0M0 n=97 (24.4)		≤6 (n = 44)	0 to <4: n=13 (5.1%)	0 to <4: n=161 (89.3%)	Mean ± SD (n=255)21.2 ± 6.7 95% CI (20.4 to 22)	Mean ± SD (n= 255)12.1 ± 6.4 95% CI (11.3 to 12.9)	− 9.1 ± 7.3, (n=255). Mean Difference ‐9.100, Standard error 0.580, 95% CI (‐10.2399 to ‐7.9601), t‐statistic ‐15.683, P < 0.0001	Intramuscular injection 15mg Triptorelin pamoate (once every 3 months)/12 weeks	48
							T4N0M0 n=10 (2.5)		7 (n = 116)	≥4 to 10: n=14 (5.5%)	≥4 to 10: n=8 (4.4%)					
							T(any)N(any)M+ n=168 (42.2)		≥8 (n = 181) from sample of n=341	≥10: n=226 (89.3%) from sample n=253	≥10: n=21 (11.7%) from sample n=180					
							Regional lymph nodes status (N+) n=22 (5.5)									
							Other (TNM stage not re‐evaluated for disease recurrence after radical treatment) n=101 (25.4)									
				0			Missing data 0		Missing data Unclear	Missing data 2	Missing data 0					
Alexandre Peltier, 2015, Belgium	Cohort (prospective)	325	161	164	73.0 years	Missing	TNM staging: N	TNM staging: M	≤6 (n = 106)	Median PSA level 10.3 ng/mL (range: 0 to 4400 ng/mL, n=164), 19 patients with moderate or severe LUTS had a PSA level <4 ng/mL	Median PSA 0.1 ng/mL (range: 0 to 137 ng/mL, n= 143)	Mean ± SD (n= 164)14.0 ± 5.3 95% CI (13.2 to 14.8)	Mean ± SD (n= 137)9.8 ± 5.1 95% CI (8.95 to 10.7)	−4.2, (n= 137) Mean Difference ‐4.200, Standard error 0.603, 95% CI (‐5.3867 to ‐3.0133), t‐statistic ‐6.965, P < 0.0001	4 injections of 11.25mg, one every 12 weeks, or 12 injections of 3.75mg, one every 4 weeks	48
							N0 178 (57.1%)	M0 199 (63.4%)	7 (n = 99)							
							N1 43 (13.8%)	M1 35 (11.1%)	≥8 (n = 94)							
							NΧ 91 (29.2%)	M1 28 (8.9%)	Missing data 26							
								M1a 1 (0.3%)								
								M1b 6 (1.9%)								
								MΧ 80 (25.5%)								
							Missing data 13	Missing data 11		Missing data Unclear	Missing data Unclear					
Henry Woo, 2017, Australia	Cohort (prospective)	44	39	5	75.6 years	79.8 kg			≤6 (n = 2)	0 to <4: n=1 (2.9%)	The mean change ± SD from baseline to week 48:‐51.6±129.8 ng/mL (median ‐12.4, n=21, p=0.0838)	Mean ± SD (n= 39)16.1±6.5 95% CI (14.1 to 18.1)	Mean ± SD (n= 23)10.5±5.8 95% CI (8.13 to 12.9)	‐5.6, (n=23) Mean Difference ‐5.600, Standard error 1.644, 95% CI (‐8.8881 to ‐2.3119), t‐statistic ‐3.407, P = 0.0012	11.25 mg (as embonate salt): 4 intramuscular injections of triptorelin 11.25 mg, according to a schedule of 1 injection administered every 12 weeks	48
							No reported data		7 (n = 13)	≥4 to 10: n=9 (25.7%)						
									≥8 (n = 14)	≥10: n=25 (71.4%) from sample n=35						
							Missing data 39		Missing data 10	Missing data 4	Missing data Unclear					

### 3.2 Meta‐analysis

**FIGURE 2:**
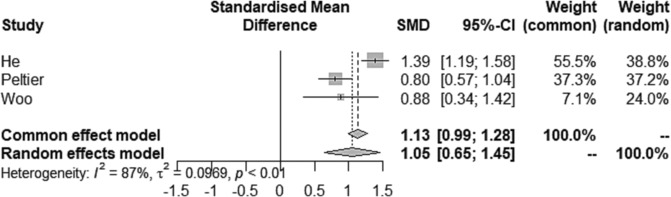
Forest plot: the effects of triptorelin therapy on IPSS change from baseline to 48 weeks therapy. Heterogeneity: 𝜏2 = 0.0969; I^2^ = 87%; (*P* < 0.01). 95% CI, confidence intervals; SMD, standard measure values.

A random‐effects model was selected to generate a forest plot (Figure 2). Forest plots in Figure 2 display results of the meta‐analysis for the effect of triptorelin therapy on IPSS change. Of the three studies analysed, all were above ‘the line of no difference’. The line of no difference is at 0 (95% CI), and the pooled effect size is a modest 1.0497 (95% CI: 0.65; 1.45, z = 5.16, *P* < 0.0001) for the random‐effects model. The forest plot suggests that the intervention of triptorelin is 1.05 times better in reducing the standardised mean difference in LUTS score compared to baseline. As the effect size is positive (>0), the results are found to be statistically significant. He et al. contributed 38.8% to the pooled effect, followed by Peltier et al. contributing 37.2% and Woo et al. contributing 24.0%.

Quantifying heterogeneity by 𝜏^2^ and *I* ^2^: 𝜏^2^ = 0.0969 (95% CI: 0.01; 3.92); 𝜏 = 0.3113 (95% CI: 0.11; 1.98). *I* ^2^ = 86.6% (95% CI: 61.4%; 95.3%); H = 2.73 (95% CI: 1.61; 4.63). Test of heterogeneity: Q = 14.90, 2 degrees of freedom, *P* = 0.0006 (*P* < 0.01), shows a statistically significant finding, that is, as the ‘random effects’ diamond shape does not touch the line of no effect, the difference between baseline and 48 weeks is statistically significant. The *I* ^*2*^ = 87% statistic indicates heterogeneity, that is, is above 50%, supporting selection of a random effects model.

**FIGURE 3:**
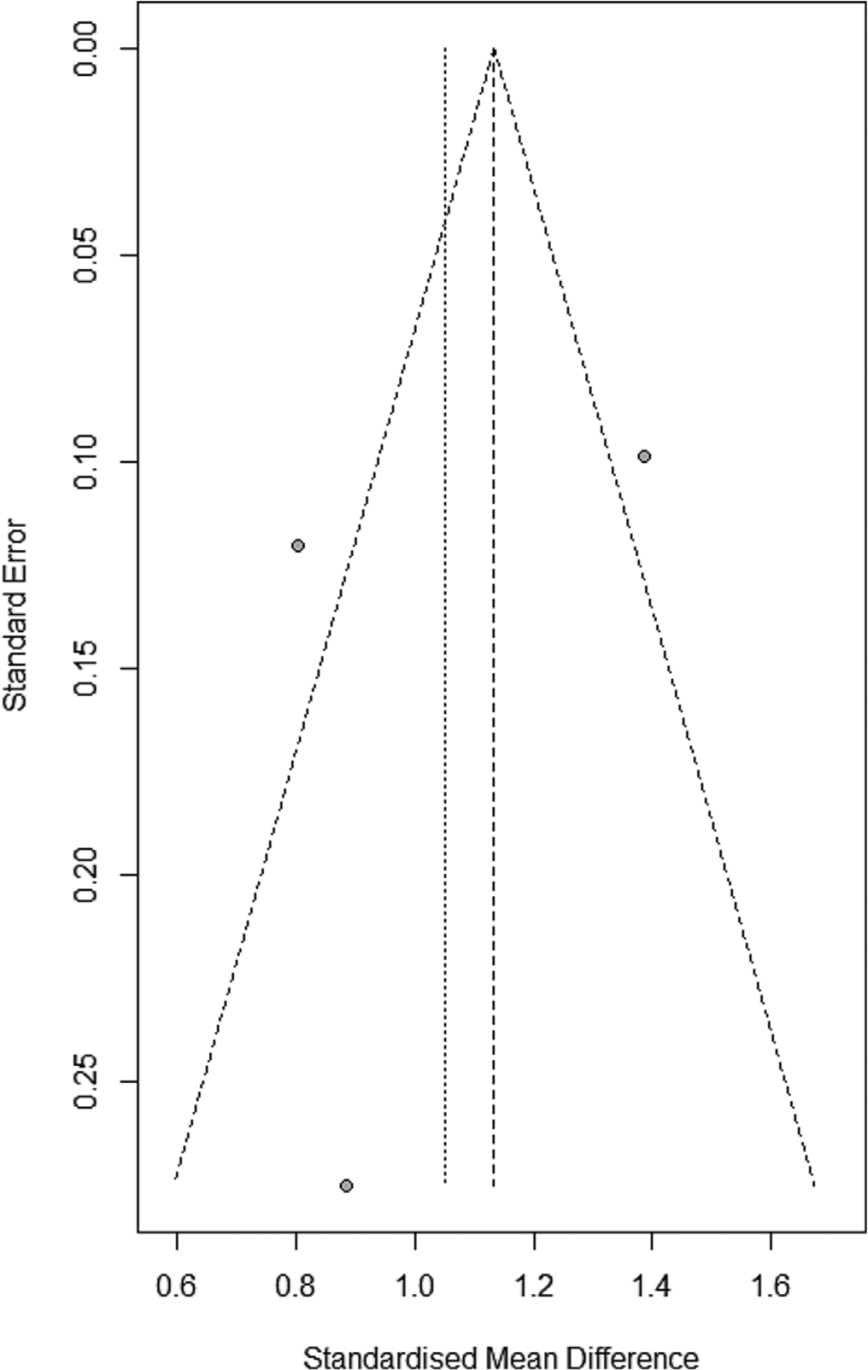
Funnel plot.

Funnel plot in Figure 3 indicates a degree of publication bias, which is supported through risk of bias assessment.

### 3.3 IPSS

The observed changes in IPSS score from baseline to Week 48 for all included studies were at least −4.2 and are outlined in Table 1.

### 3.4 QoL

All included studies assessed changes in patient QoL using the last question of the IPSS: ‘If you were to spend the rest of your life with your urinary condition just the way it is now, how would you feel about that?’.^44^ QoL was measured using a 7‐point Likert scale ranging from 0 (*delighted*), 1 (*pleased*), 2 (*mostly satisfied*), 3 (*equally satisfied and dissatisfied*), 4 (*mostly dissatisfied*), 5 (*unhappy*), to 6 (*terrible*).

In Table 2, Peltier et al. provided clear QoL values. He et al. provided sufficient evidence for meta‐analysis authors to calculate the mean scores shown. Woo et al. indicated that 38 out of 44 patients had a score from 0 to 2 at baseline indicating a relatively good baseline and 22 of the 23 patients had a score from 0 to 2 at Week 48, with several patients lost to follow‐up, making change in QoL assessment unclear. However, overall, the data suggest that there is improvement in patients' QoL.

**TABLE 2:** The mean QoL score assessing urinary symptoms at baseline and at Week 48 for PCa patients taking triptorelin (a reduction in score indicates an improvement in QoL).

Author (year)	Mean QoL score at baseline	Mean QoL score at week 48	Change in IPSS QoL from baseline to week 48
He et al. (2018)	4.3 (*n* = 255)	2.3 (*n* = 194)	−1.9
Peltier et al. (2015)	2.9 ± 1.1 (*n* = 164)	2 (*n* = 137)	−0.9 ± 1.3 (*P* < 0.05)
Woo et al. (2017)	0–2 (*n* = 44)	0–2 (*n* = 23)	Unclear

### 3.5 Risk of bias quality assessment of included studies

Risk of bias was assessed using the NOS and is summarised in Table 3 under headings for selection bias, outcome bias and total bias.

**TABLE 3:** The Newcastle–Ottawa Scale (NOS) used to assess the quality of included nonrandomised studies in this review.

Author	Selection	Outcome	Total
He et al. (2018)	★★★	★★★	6/9
Peltier et al. (2015)	★★★	★★★	6/9
Woo et al. (2017)	★★★	★★	5/9

All studies receive a scored 3 out of 4 in the ‘selection’ bias criteria because they did not include a control arm and therefore did not receive this full score. However, this did not bias the review, as all studies were observational and non‐interventional, with appropriate patient follow‐up. No study, received a score on the ‘comparability’ criteria, as no control arm existed. He et al. and Peltier et al. received a score of 3 in the ‘outcome’ criteria. Although both studies had a drastically reduced full analysis population (>15%) when compared to the study population, these studies accounted for every patient not included in the full analysis population. Woo et al. failed to account for every patient that was not included in the full analysis population and thus did not receive a score regarding adequacy of follow up. From this assessment, this meta‐analysis is judged to be at a ‘moderate to high risk of bias’. The full bias assessment is shown in Data S1 on NOS.

Sources of funding were fully declared:

- Woo et al. Trials Registry (ACTRN12610000558022) was queried; however, summary results were not documented: https://www.anzctr.org.au/Trial/Registration/TrialReview.aspx?id=335621&isReview=true. Financial support was received from Ipsen Pty Ltd., Australia. Authors report receiving honoraria, research grant and being employees of Ipsen Pty Ltd.
- Peltier et al. No financial interest or financial conflict noted, nor financial support received. Though one author was an employee of Ipsen NV, Belgium. Some argue this introduces bias. However, as these companies are involved in direct drug development and research of triptorelin, their participation in such publication is expected. Trial identifier I‐48‐52014‐150 was searched for on the Federal Agency for Medicines and Health Products in Belgium and under EU clinical trials register but could not be retrieved.
- The He et al. study and analysis was funded by Ipsen, who also provided writing assistance in manuscript preparation.

## 4 DISCUSSION

The studies included in this meta‐analysis showed improvement in LUTS in men with PCa over 48 weeks of triptorelin therapy due to the clinically significant IPSS changes from baseline (a 3‐point reduction in IPSS). A random effects model shows a pooled effect size is 1.05 (95% CI: 0.65; 1.45, z = 5.16, *P* < 0.0001), suggesting significant benefits accrued to patients. Specifically, there was a reduction in urinary frequency, urgency, nocturia and improved peak urinary flow rate. However, there was considerable heterogeneity among the studies regarding the outcome measures used. The quality assessment revealed that the included studies had a moderate to high risk of bias.

Findings suggest that the effects of triptorelin therapy on LUTS are comparable across different countries. However, there has been no direct comparison of triptorelin against other LH‐RH agonists with respect to efficacy in improving LUTS in men with PCa. Previous studies^10^, ^38^ looking at the effect of LH‐RH agonists on LUTS and carried out for 12 weeks demonstrated improvements. The studies used in this meta‐analysis had a longer follow‐up period (48 weeks) showing that the effects of LH‐RH agonists on LUTS are sustained over a clinically relevant period. A longer follow‐up allows for treatment‐emergent adverse effects, patient satisfaction and full therapeutic effects to emerge, which can only happen after sustained use.^45^ The benefit of these observational studies is that they are conducted in the real‐world clinical setting giving an indication on the impact of triptorelin therapy in reducing LUTS in the PCa patient population.

All included studies reported clinically meaningful reductions in IPSS in men with both PCa and moderate to severe LUTS treated with triptorelin. LH‐RH agonists have been recognised to significantly reduce the size of the prostate gland. Therefore, assuming that the urinary symptoms associated with PCa are most likely due, at least in part, if not wholly to outflow obstruction, the response to triptorelin therapy would not be surprising.

It must be remembered that LUTS arise from a variety of causes. What is described here is a specific subset of men with both PCa and moderate to severe LUTS, and it is within this population that the results of the presented meta‐analysis must be interpreted. As a result, this meta‐analysis does not reflect the broader use of the term LUTS in a variety of other settings.

So why is triptorelin important in post‐pandemic practice? The pandemic has had an impact on how clinical care is now being delivered. Because of it, now, there exist backlogs for patients seeking PCa consultation and interventions in the United Kingdom as well as globally. The changes to how care was delivered during the pandemic are also sustained, that is, use of longer acting formulations being routinely used, making this paper and triptorelin important in current clinical care decisions. The frequency and severity of adverse events (hot flushes, fatigue, sexual dysfunction) appear to be comparable among LH‐RH agents. In already published research,^46^ it is evident that since the pandemic, changes have appeared in how prescription LH‐RH medications have been used in the United Kingdom. Prescription volumes for all LH‐RH agents were much lower initially (January 2109 to October 22), but evidence of switching towards the 6‐month (24 weeks) preparation offered by triptorelin is clear. Triptorelin is the only LH‐RH agent with a 24‐week formulation that allows it to be used in this way.

While the switch was observed during the pandemic to reduce clinical contact time, improved prescription day coverage and to reassure patients of treatment continuity, it remains unclear whether these patients will switch back to a formulation requiring more frequent administration. From a medicines management perspective, this seems unlikely and unnecessary if they are stabilised on these therapies without burdensome adverse effects. Of note, triptorelin comes in several different salt formulations such as acetate, pamoate and embonate, which may allow easier in‐agent transfer/switching, improving choice.

Treatment‐related adverse drug reactions may emerge from goserelin, leuprorelin or triptorelin but are unlikely to be significantly different because of the similarities of their mechanism of action, as is supported by literature.^47^, ^48^ However, goserelin has been on the market for over 40 years (compared to 2002 UK authorisation for triptorelin, i.e. 21 years, and 2011 for leuprorelin, i.e. 12 years). This does not diminish the impact on the QoL and patient experiences of using these agents. Experience, clinician acceptability and patient satisfaction will also influence clinicians' choice of agent. The result of this paper may aid that process. Alternatives to therapy can include surgical options: Managing LUTS involves a comprehensive approach that may include lifestyle modifications, behavioural therapies, medications and in some cases, surgical interventions. However, some men with PCa may be reluctant to consider surgical options and have failed treatment with oral agents, especially post‐pandemic.

Future studies are important to consider. Cost effectiveness and budget impact analysis remain under studied in the wider healthcare literature for these agents. Pharmacoeconomic or health economic analysis is prevented because all costs associated with alternative surgical interventions like TURP in comparison to the average monthly cost of LH‐RH agents like triptorelin are not fully quantified. The monthly cost of triptorelin in the United Kingdom (~£8.03 million for 47 632 doses/month = £168.58 m in Oct 2020; see Data S1, ^46^) has been previously published.^46^

Access to the individual patient data in an anonymised format from each of the three studies would be useful (this could be published as a supplement or deposited in a free public repository like Open Science Framework or GitHub) allowing secondary analysis, which would be more mathematically robust in a meta‐analysis. Authors of this meta‐analysis would be happy to reanalyse this data, in addition to new data.

Increasingly genetic and genomic therapies offer precision medicine treatment options, which have not been considered here.

There are several strengths in the presented meta‐analysis as all included studies had a 48‐week follow‐up, which is clinically relevant. However, this has to be balanced with patient acceptability and the burden of clinic visits as well as injection use. The use of IPSS as a validated scale increases the validity and generalisability of the individual studies and adds credibility to this review. Reporting across studies is clear and has been presented in a relatively standard way, allowing for a valid review. Each study tended to be multicentre based increasing the generalisability and better reflecting routine clinical practices rather than strictly controlled experimental environments.

There are several limitations to this meta‐analysis, which include the low sample size, lack of power calculations, likely less than 80% (not reported) and large standard errors. There was also a lack of ethnic diversity in the patient base. Formulation salts were not reported accurately, for example, acetate, embonate and pamoate were not mentioned each time, and this meta‐analysis assumes that they were not used interchangeably.

The included studies did not include patients with mild LUTS symptoms in the full analysis population. Demographics, for example, weight, and concomitant medicines were not fully reported, limiting direct comparison, and this meta‐analysis relies on the individual study's principal investigators to have made conservative and representative choices of their patient population. The IPSS is not an objective measure, but a proxy for the measurement of LUTS as it relies on subjective recall by patients, that is open to bias.

A further limitation of this review is that the ‘quality of life’ appraisal was restricted to responses to one question. Other aspects that influence quality of life such as adverse drug reactions to ADT, or the negative impacts of low testosterone including fatigue, weight gain, reduced cognitive function and reduced muscle mass, as a function of lower testosterone levels, are all negative impacts that would appear as consequences of ADT therapy in any case. Bear in mind these patients were on triptorelin for ADT, not for LUTS.

Inherent limitations included a lack of randomisation and lack of a control group due to the observational nature of the studies included. The included studies failed to recruit the intended number of patients. Furthermore, this meta‐analysis did not consider other medications patients that may have been prescribed while on triptorelin therapy. The use of such co‐mediations may have impacted changes in LUTS severity and the reproducibility of the results. What has not been studied is the impact of cystitis, unary tract infections, benign prostatic hyperplasia or information related to surgical procedures in and around the prostate gland.

The patient's quality of life is an important consideration. The burden of LUTS in prostate cancer is expected to rise with the ageing population, leading to potential social and economic implications in the future. Addressing the increasing prevalence of LUTS requires effective and cost‐efficient management strategies. Triptorelin has shown promise in improving LUTS in men with PCa and can play a significant role in managing this condition in this subset of patients.

Triptorelin treatment has been found to have a positive impact on the QoL of men with PCa, urinary symptoms. This improvement in QoL has several positive implications. Firstly, it can contribute to increased productivity by enabling patients to continue working or engage in daily activities without the burden of bothersome urinary symptoms. This, in turn, could benefit the overall economy by maintaining a productive workforce. While many of the patient populations in this study may have retired, this does not mean they are not supporting economically viable activities that are (un/)remunerated or supporting their wider family in making financial or inheritance decisions. These men may be retired, but still have a contribution to make to the economy either directly or indirectly.

Furthermore, the enhancement of LUTS related QoL through triptorelin treatment can have positive effects on patient well‐being and satisfaction with their treatment. Improved QoL is associated with better physical and mental health outcomes, leading to overall improved patient health and outcomes. When patients experience a higher QoL, they are more likely to adhere to their treatment plan, engage in self‐care and actively participate in their healthcare decisions.

In conclusion, triptorelin appears to have a positive impact on LUTS in men with prostate cancer. Studies included in this meta‐analysis reported clinically significant reductions in mean IPSS in PCa patients with moderate to severe LUTS who received triptorelin therapy. However, the heterogeneity among the studies and the ‘moderate to high’ risk of bias means findings should be interpreted with caution.

## AUTHOR CONTRIBUTIONS

Ravina Barrett led this meta‐analysis and review. Ravina Barrett conducted the literature search, study conception and design, data analysis, statistical analysis, including meta‐analysis and interpretation of data, manuscript preparation, editing and revision, responding to reviewers, creating a PRISMA checklist and submitted the final version of the paper. Ravina Barrett generated all the supplemental materials, syntax, tables and figures. Ravina Barrett supervised FO during their research project (at Level 7). Brian Birch provided critical intellectual review and considered the clinical impact and consequences of findings on this patient population. All authors agreed on the final manuscript for publication. Collaborators: FO conducted the preliminary literature search, brief data synthesis (without meta‐analysis) and prepared the draft manuscript, which was submitted as part of their research thesis for MPharm award. They have not contributed to the design of this review, nor to the final write‐up and publication.

## CONFLICT OF INTEREST STATEMENT

RB and BB (authors) have completed the ICMJE uniform disclosure form at www.icmje.org/coi_disclosure.pdf and declare: no financial relationships or activities that could appear to have influenced the submitted work. No financial or non‐financial support was received for this review. This Meta‐Analysis was independently conducted during RB's employment and did not receive any specific funding/expertise from external sources.

## REGISTRATION AND PROTOCOL

This meta‐analysis was not registered. A protocol was not prepared.

## SPONSOR

The sponsor of this Meta‐Analysis is the University of Brighton.

## Supporting information


**Data S1.** Supporting Information.

**Data S2.** Supporting Information.

**Data S3.** Supporting Information.

**Data S4.** Supporting Information.

## Data Availability

Data extracted from included studies are presented in this meta‐analysis and in its supplementary material. No further material was used in this review. There is no further availability of data, code or other materials.
