# Supplementary material for: Triptorelin therapy for lower urinary tract symptoms (LUTS) in prostate cancer patients: A systematic meta‐analysis
Source: BJUI Compass. 2023 Oct 10;5(1):17–28. doi: 10.1002/bco2.292 (PMC10764163; doi:10.1002/bco2.292)
Supplement: Supplementary file 1 — Data S1. Supporting Information. [file BCO2-5-17-s003.docx]

## **Supplementary Material 1**: Search Term and Results

### PubMed:

Search: **((PCa patient) AND (luteinising hormone releasing hormone agonist)) AND (lower urinary tract symptoms)** Sort by: **First Author**

Full search:

("prostatic neoplasms"[MeSH Terms] OR ("prostatic"[All Fields] AND "neoplasms"[All Fields]) OR "prostatic neoplasms"[All Fields] OR ("prostate"[All Fields] AND "cancer"[All Fields]) OR "PCa"[All Fields]) AND ("patient s"[All Fields] OR "patients"[MeSH Terms] OR "patients"[All Fields] OR "patient"[All Fields] OR "patients s"[All Fields]) AND (("gonadotropin releasing hormone"[MeSH Terms] OR ("gonadotropin releasing"[All Fields] AND "hormone"[All Fields]) OR "gonadotropin releasing hormone"[All Fields] OR ("luteinising"[All Fields] AND "hormone"[All Fields] AND "releasing"[All Fields] AND "hormone"[All Fields]) OR "luteinising hormone releasing hormone"[All Fields]) AND ("agonist"[All Fields] OR "agonist s"[All Fields] OR "agonistic"[All Fields] OR "agonistically"[All Fields] OR "agonistics"[All Fields] OR "agonists"[MeSH Subheading] OR "agonists"[All Fields])) AND ("lower urinary tract symptoms"[MeSH Terms] OR ("lower"[All Fields] AND "urinary"[All Fields] AND "tract"[All Fields] AND "symptoms"[All Fields]) OR "lower urinary tract symptoms"[All Fields])

### Web of Science

**(((((ALL=(PCa )) AND ALL=(lower urinary tract symptoms )) AND ALL=(luteinizing hormone releasing hormone agonist )) OR ALL=(triptorelin)))**

**Refined by: IPSS (all fields)**

### Embase

**Pca [title], lower urinary tract symptoms [title], luteinising hormone releasing hormone agonist or triptorelin in EMBASE.**

| Search number | Search term |
| --- | --- |
| 4 | Limit 3 to (full text and human and english language and yr="2013 - 2023") |
| 5 | lower urinary tract symptoms.m_titl. |
| 6 | PCa.m_titl. |
| 7 | (luteinising hormone releasing hormone agonist or triptorelin).mp. [mp=title, abstract, heading word, drug trade name, original title, device manufacturer, drug manufacturer, device trade name, keyword heading word, floating subheading word, candidate term word] |

Full search: 4 and 5 and 6 and 7

## **Supplementary Material 2:** Calculated Mean Age of Study Participants.

| Author | Year | Region | Mean age (years) |
| --- | --- | --- | --- |
| Le-Ye He | 2018 | China | 72.2 |
| Alexandre Peltier | 2015 | Belgium | 73.0 |
| Henry Woo | 2017 | Australia | 75.6 |
| Total mean age of all included studies |  |  | 73.6 |

## **Supplementary material 3:** The Newcastle-Ottawa Scale (NOS) used to assess the quality of the 3 studies included in the systematic review.

**NOS scale: He et al. (2018)**

Note: A study can be awarded a maximum of one star for each numbered item within the Selection and Outcome categories. A maximum of two stars can be given for Comparability.

| **No.** | **Criterion** | **Decision rule** | **Score (*=1, no*=0)** |
| --- | --- | --- | --- |
| **SELECTION** | | | |
| 1 | Representativeness of the exposed cohort | 1. Consecutive eligible participants were selected, participants were randomly selected, or all participants were invited to participate from the source population* 2. Not satisfying requirements in part (a), or not stated. | ***** |
| 2 | Selection of the non-exposed cohort | 1. Selected from the same source population* 2. Selected from a different source population 3. No description |  |
| 3 | Ascertainment of exposure | 1. Structured injury data (e.g. record completed by medical staff)* 2. Structured interview* 3. Written self-report 4. No description | ***** |
| 4 | Demonstration that outcome of interest was not present at  the start of the study | 1. Yes* 2. No or not explicitly stated | * |
| **COMPARABILITY** | | | |
| 1 | Comparability of cohorts on the basis of the design or analysis | 1. Study controls for previous injury* 2. Study controls for age*   *Note:* Exposed and non-exposed individuals must be matched in the design and/or confounders must be adjusted for in the analysis. Alone statements of no differences between groups or that differences were not statistically significant are not sufficient. |  |
| **OUTCOME** | | | |
| 1 | Assessment of outcome | 1. Independent or blind assessment stated, or confirmation of the outcome by reference to secure records (e.g. imaging, structured injury data, etc.)* 2. record linkage (e.g. identified through ICD codes on database records)* 3. Self-report with no reference to original structured injury data or imaging 4. No description | * |
| 2 | Was follow-up long  enough for outcomes to occur? | 1. Yes (≥3 months)* 2. No (<3 months) | * |
| 3 | Adequacy of follow up of cohorts | 1. Complete follow up – all participants accounted for* 2. Subjects lost to follow up unlikely to introduce bias (<15% lost to follow up, or description provided of those lost*) 3. Follow up rate <85% and no description of those lost provided 4. No statement | * |
|  | | **SCORE:** | 6 / 9 |

**NOS scale: Peltier et al. (2015)**

Note: A study can be awarded a maximum of one star for each numbered item within the Selection and Outcome categories. A maximum of two stars can be given for Comparability.

| **No.** | **Criterion** | **Decision rule** | **Score (*=1, no*=0)** |
| --- | --- | --- | --- |
| **SELECTION** | | | |
| 1 | Representativeness of the exposed cohort | 1. Consecutive eligible participants were selected, participants were randomly selected, or all participants were invited to participate from the source population* 2. Not satisfying requirements in part (a), or not stated. | ***** |
| 2 | Selection of the non-exposed cohort | 1. Selected from the same source population* 2. Selected from a different source population 3. No description |  |
| 3 | Ascertainment of exposure | 1. Structured injury data (e.g. record completed by medical staff)* 2. Structured interview* 3. Written self-report 4. No description | ***** |
| 4 | Demonstration that outcome of interest was not present at  the start of the study | 1. Yes* 2. No or not explicitly stated | * |
| **COMPARABILITY** | | | |
| 1 | Comparability of cohorts on the basis of the design or analysis | 1. Study controls for previous injury* 2. Study controls for age*   *Note:* Exposed and non-exposed individuals must be matched in the design and/or confounders must be adjusted for in the analysis. Alone statements of no differences between groups or that differences were not statistically significant are not sufficient. |  |
| **OUTCOME** | | | |
| 1 | Assessment of outcome | 1. Independent or blind assessment stated, or confirmation of the outcome by reference to secure records (e.g. imaging, structured injury data, etc.)* 2. record linkage (e.g. identified through ICD codes on database records)* 3. Self-report with no reference to original structured injury data or imaging 4. No description | * |
| 2 | Was follow-up long  enough for outcomes to occur? | 1. Yes (≥3 months)* 2. No (<3 months) | * |
| 3 | Adequacy of follow up of cohorts | 1. Complete follow up – all participants accounted for* 2. Subjects lost to follow up unlikely to introduce bias (<15% lost to follow up, or description provided of those lost*) 3. Follow up rate <85% and no description of those lost provided 4. No statement | * |
|  | | **SCORE:** | 6 / 9 |

**NOS scale: Woo et al. (2017)**

Note: A study can be awarded a maximum of one star for each numbered item within the Selection and Outcome categories. A maximum of two stars can be given for Comparability.

| **No.** | **Criterion** | **Decision rule** | **Score (*=1, no*=0)** |
| --- | --- | --- | --- |
| **SELECTION** | | | |
| 1 | Representativeness of the exposed cohort | 1. Consecutive eligible participants were selected, participants were randomly selected, or all participants were invited to participate from the source population* 2. Not satisfying requirements in part (a), or not stated. | ***** |
| 2 | Selection of the non-exposed cohort | 1. Selected from the same source population* 2. Selected from a different source population 3. No description |  |
| 3 | Ascertainment of exposure | 1. Structured injury data (e.g. record completed by medical staff)* 2. Structured interview* 3. Written self-report 4. No description | ***** |
| 4 | Demonstration that outcome of interest was not present at  the start of the study | 1. Yes* 2. No or not explicitly stated | * |
| **COMPARABILITY** | | | |
| 1 | Comparability of cohorts on the basis of the design or analysis | 1. Study controls for previous injury* 2. Study controls for age*   *Note:* Exposed and non-exposed individuals must be matched in the design and/or confounders must be adjusted for in the analysis. Alone statements of no differences between groups or that differences were not statistically significant are not sufficient. |  |
| **OUTCOME** | | | |
| 1 | Assessment of outcome | 1. Independent or blind assessment stated, or confirmation of the outcome by reference to secure records (e.g. imaging, structured injury data, etc.)* 2. record linkage (e.g. identified through ICD codes on database records)* 3. Self-report with no reference to original structured injury data or imaging 4. No description | * |
| 2 | Was follow-up long  enough for outcomes to occur? | 1. Yes (≥3 months)* 2. No (<3 months) | * |
| 3 | Adequacy of follow up of cohorts | 1. Complete follow up – all participants accounted for* 2. Subjects lost to follow up unlikely to introduce bias (<15% lost to follow up, or description provided of those lost*) 3. Follow up rate <85% and no description of those lost provided 4. No statement |  |
|  | | **SCORE:** | 5 / 9 |
