# Supplementary material for: Triptorelin therapy for lower urinary tract symptoms (LUTS) in prostate cancer patients: A systematic meta‐analysis
Source: BJUI Compass. 2023 Oct 10;5(1):17–28. doi: 10.1002/bco2.292 (PMC10764163; doi:10.1002/bco2.292)
Supplement: Supplementary file 2 — Data S2. Supporting Information. [file BCO2-5-17-s002.pdf]

```r
###   Systematic Review and Meta-Analysis on the Effects of Triptorelin on Lower Urinary Tract Symptoms (LUTS) in Patients with Prostate Cancer

###   BCO2-2023-074 [email ref: DL-RW-3-a]



install.packages("meta")
library(meta)
Meta <- read.csv("C:/Users/rb1097/OneDrive - University of Brighton/PYM40/Funmi Olugbodi/Manuscript/Meta.csv")
View(Meta)
head(Meta)


m1 <- metacont (n.Baseline, Mean.Baseline,SD.Baseline,
               n.At48Wks, Mean.At48Wks, SD.At48Wks,
               fixed=T, random=T, studlab=ï..Author,
               data=Meta, sm="SMD")

m1

#Number of studies: k = 3
#Number of observations: o = 873

#                        SMD            95%-CI     z  p-value
#Common effect model  1.1334 [0.9893; 1.2776] 15.41 < 0.0001
#Random effects model 1.0497 [0.6508; 1.4486]  5.16 < 0.0001

#Quantifying heterogeneity:
# tau^2 = 0.0969 [0.0128; 3.9230]; tau = 0.3113 [0.1132; 1.9807]
# I^2 = 86.6% [61.4%; 95.3%]; H = 2.73 [1.61; 4.63]

#Test of heterogeneity:
#     Q d.f. p-value
# 14.90    2  0.0006

#Details on meta-analytical method:
#- Inverse variance method
#- Restricted maximum-likelihood estimator for tau^2
#- Q-Profile method for confidence interval of tau^2 and tau


forest(m1, leftcol=c('studlab'))
funnel(m1)
```
